# OCTN2- and ATB^0,+^-targeted nanoemulsions for improving ocular drug delivery

**DOI:** 10.1186/s12951-024-02402-x

**Published:** 2024-03-26

**Authors:** Bo Tang, Qiuxiang Wang, Guowei Zhang, Aiwen Zhang, Lu Zhu, Rongrong Zhao, Hongwei Gu, Jie Meng, Junfang Zhang, Guihua Fang

**Affiliations:** 1 School of Pharmacy, Nantong University, 19 Qixiu Road, Nantong, 226001 Jiangsu China; 2 Eye Institute, Affiliated Hospital of Nantong University, Nantong, 226001 Jiangsu China

**Keywords:** Stearoyl l-carnitine, Nanoemulsions, Novel organic cation/carnitine transporter 2 (OCTN2), Amino acid transporter B (0 +) (ATB^0,+^), Endotoxin-induced uveitis, Ocular drug bioavailability

## Abstract

Traditional eye drops are administered via topical instillation. However, frequent dosing is needed due to their relatively rapid precorneal removal and low ocular bioavailability. To address these issues, stearoyl L-carnitine-modified nanoemulsions (SC-NEs) were fabricated. The physicochemical properties of SC-NEs in terms of size, morphology, zeta potential, encapsulation efficiency, and in vitro drug release behavior were characterized. The cellular uptake and mechanisms of SC-NEs were comprehensively studied in human corneal epithelial cells and the stearoyl L-carnitine ratio in SC-NEs was optimized. The optimized SC-NEs could target the novel organic cation/carnitine transporter 2 (OCTN2) and amino acid transporter B (0 +) (ATB^0,+^) on the corneal epithelium, which led to superior corneal permeation, ocular surface retention ability, ocular bioavailability. Furthermore, SC-NEs showed excellent in vivo anti-inflammatory efficacy in a rabbit model of endotoxin-induced uveitis. The ocular safety test indicated that the SC-NEs were biocompatible. In general, the current study demonstrated that OCTN2 and ATB^0,+^-targeted nanoemulsions were promising ophthalmologic drug delivery systems that can improve ocular drug bioavailability and boost the therapeutic effects of drugs for eye diseases.

**Graphical Abstract:**

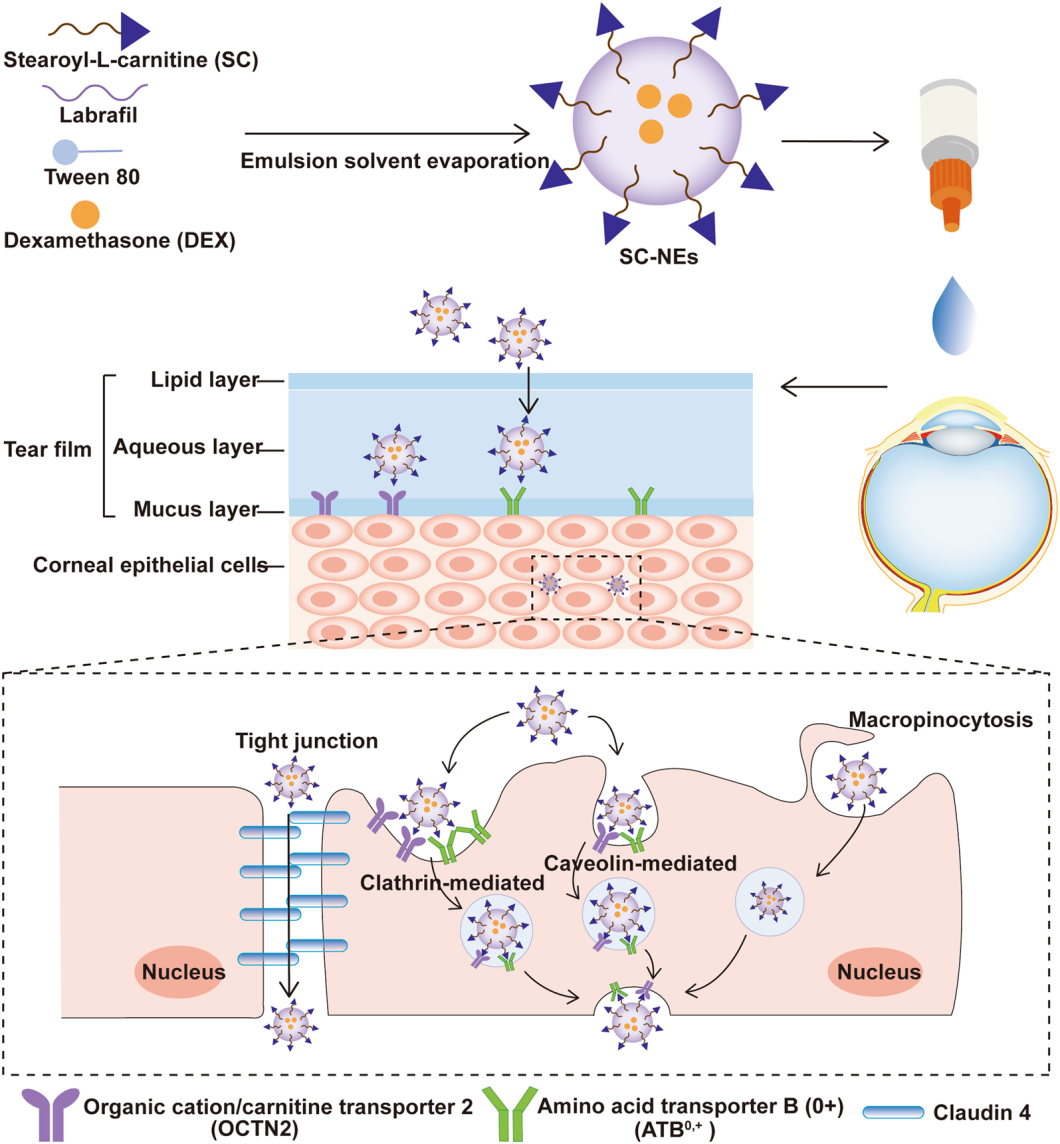

**Supplementary Information:**

The online version contains supplementary material available at 10.1186/s12951-024-02402-x.

## Introduction

Topical instillation of eye drops is the most common approach for the treatment of ophthalmic diseases [1, 2]. However, conventional eye drops show poor ocular bioavailability (< 5%) owing to the eye anatomy and physiology, such as blinking and lachrymation, which greatly decreases the ocular surface retention time and reduces the corneal permeability of drugs [3]. One tactic to address these issues is to increase the frequency and dose of administration, which reduces the patient patience and has the risk of side effects [4]. Another tactic is to extend the ocular surface retention time and promote corneal permeability, which has been proven to be a highly effective way to improve ocular bioavailability [5–7].

Nanotechnology has provided great opportunities for improving ocular drug delivery over the last decades [8]. Nanotechnology-based ocular drug delivery systems, such as liposomes, nanoemulsions, nanoparticles, and micelles can increase the solubility of poorly water-soluble drugs and improve ocular surface retention time as well as corneal permeability, thus increasing ocular bioavailability [9, 10]. Among these various drug delivery systems, nanoemulsions (NEs), well-developed lipid-based colloidal dispersions, have been extensively used to treat various ophthalmic diseases (e.g., glaucoma, dry eye syndrome, and age-related macular degeneration) because of their easy preparation, high drug loading capacity, and cost-effective [11–13]. Moreover, there are several commercially available ophthalmic NEs (e.g., Restasis®, Cationorm®, Ikervis®) have been approved for human use. NEs can improve ocular drug bioavailability by extending precorneal retention time and increasing corneal permeability across tight junctions [14]. However, NEs alone still cannot deliver a sufficient amount of drug into the intraocular tissues and maintain their therapeutic efficacy for a longer time. Recent studies have demonstrated that the surface of nanocarrier modification with ligands can further facilitate mucosa adhesion and enhance corneal permeability by ligand-receptor interactions, thus improving ocular drug bioavailability. Some ligands, such as penetratin [13], cyclic peptide cRGD [15], and hyaluronic acid [16] have been employed to modify nanocarriers for fabricating targeted ocular delivery systems, and these targeted nanocarriers exhibited better ocular delivery efficiency in vivo compared with untargeted nanocarriers. Therefore, NEs modified with a ligand have great potential to further improve the ocular bioavailability of drugs.

L-carnitine, an endogenous polar compound, can transfer long-chain aliphatic acid into mitochondria for degradation through β-oxidation, and thus the cells obtain energy from the stored fat [17]. L-carnitine uptake into cells was regulated by the novel organic cation/carnitine transporter 2 (OCTN2) [18]. OCTN2 has a high affinity for L-carnitine and is sodium (Na^+^)-dependent and mainly responsible for L-carnitine entry into cells [19]. The Na^+^ and chloride (Cl^−^)-dependent neutral and basic amino acid transporter B (0 +) (ATB^0,+^) can also transport L-carnitine, but its affinity with L-carnitine is lower than OCTN2 [20, 21]. Both OCTN2 and ATB^0,+^ are over-expressed in the corneal epithelial cells [22, 23]. Therefore, nanoemulsions modified with L-carnitine are considered to be a promising strategy to further increase the ocular delivery efficiency of nanoemulsions via OCTN2 and ATB^0,+^-meditated corneal absorption. As far as we know, there is no relevant report on the development of nanoemulsions modified with L-carnitine targeting dual transporters (OCTN2 and ATB^0,+^) and systematic research of its application in improving ocular bioavailability.

Herein, we constructed stearoyl L-carnitine (SC)-modified nanoemulsions (SC-NEs) to target OCTN2 and ATB^0,+^ for improving ocular drug delivery. We assumed that the L-carnitine modification enabled nanoemulsions to improve the ocular surface retention time, corneal permeability, and ocular bioavailability to enhance their in vivo anti-inflammatory efficacy. First, amphiphilic stearoyl L-carnitine was synthesized, and then nanoemulsions functionalized by SC with varying modification ratios were fabricated and characterized. Cell uptake of SC-NEs in corneal epithelial cells and the mechanism of increased uptake by SC modification were elaborated. The corneal permeability and the ocular surface retention ability were also investigated. For ocular drug delivery, dexamethasone (DEX) was used as a model drug and was encapsulated in SC-NEs. The in vivo pharmacokinetics of DEX-loaded SC-NEs (DEX SC-NEs) were studied in the rabbit. The in vivo anti-inflammatory efficacy was evaluated in a rabbit model of endotoxin-induced uveitis (EIU). The ocular safety was studied in corneal epithelial cells and rabbits.

## Materials and methods

### Materials

L-carnitine and paraformaldehyde were purchased from Aladdin (Shanghai, China). Dexamethasone and coumarin 6 were purchased from Macklin (Shanghai, China). Labrafil ® M 1944 CS was kindly gifted from Gattefossé (Lyon, France). Tween 80 was purchased from BASF (Ludwigshafen, Germany). α-methyl-DL-tryptophan, primary antibody against ATB^0,+^ and OCTN2, Alexa Fluor 594 labeled secondary antibody, and rabbit anti-ATB^0,+^ antibody were purchased from Sigma-Aldrich (St. Louis, Missouri, USA). Rabbit anti-OCTN2 antibody was purchased from ABclonal (Woburn, USA). BCA kit, Cell Counting Kit-8 (CCK8), RAPI buffer, and DAPI (4’,6-diamidino-2-phenylindole) were purchased from Beyotime (Shanghai, China). Rhodamine B and endocytosis inhibitors including chlorpromazine, indomethacin, colchicine, and quercetin were purchased from Aladdin (Shanghai, China). DEX solution was prepared as follows: DEX was first dissolved in dimethyl sulfoxide (DMSO) and then the stock solution was diluted to a final DEX concentration of 0.5 mg/mL and DMSO concentration of 5% [24].

Human corneal epithelial cells (HCECs) were kindly gifted by Prof. Hongtao Song (Department of Pharmacy, 900 Hospital of the Joint Logistics Team, Fuzhou, China). HCECs were incubated in a DMEM medium including 10% FBS, 100 IU/mL penicillin, and 100 µg/mL streptomycin. The cells were incubated at 37 °C with 5% CO_2_.

New Zealand albino rabbits (2.5–3.0 kg) were purchased from the animal center of Nantong University. All the animal studies were approved by Nantong University Ethics Committee.

### Synthesis and characterization of stearoyl L-carnitine

Stearoyl L-carnitine was synthesized as described previously [25]. Details are provided in the Supporting information.

### Preparation and characterization of stearoyl L-carnitine-modified nanoemulsions (SC-NEs)

#### Preparation of SC-NEs

SC-NEs were prepared by emulsion solvent evaporation method. Briefly, the oil phase was prepared by dissolving DEX with the required amount (3 mg), Labrafil ® M 1944 CS (50 mg), Tween 80 (50 mg), and different amounts of SC in ethanol under magnetic stirring at 75 °C. The ratios of SC to the amount of Labrafil ® M 1944 CS were set as 5%, 10%, 20%, 40%, and 80%, respectively. The deionized water (5 mL) was used as an aqueous phase by heated to 75 °C. The aqueous phase was added dropwise to the oil phase under magnetic stirring at 75 °C. The resultant primary emulsions were sonicated using a probe sonicator (Scientz, China). Subsequently, the obtained nanoemulsions were centrifuged to remove the unloaded drugs. Similarly, the unmodified NEs were prepared in the same as the aforementioned procedure except without the addition of SC.

#### Characterization of SC-NEs

##### Particle size and zeta potential

The particle size and zeta potential were determined by a 90 Plus Particle Size Analyzer (Brookhaven, USA) [26]. The morphology of DEX NEs and DEX SC-NEs was visualized by a transmission electron microscope (TEM) (Hitachi, Japan) [27].

##### Encapsulation efficiency

The EE of DEX in NEs and SC-NEs was assayed by an ultrafiltration method. The unencapsulated drug was removed by ultrafiltration with an ultrafilter (MWCO: 5 kDa, Beijing Genosys). Briefly, 0.5 mL of nanoemulsions was added into an ultrafilter and centrifuged at 4000 rpm for 15 min. The DEX concentration in the filtrate was measured by HPLC. To assay the amount of drug in nanoemulsion suspensions, ethanol was placed to dissolve nanoemulsions followed by water sonication and centrifugation (13,000 rpm, 15 min). The DEX concentration in the filtrate and nanoemulsion suspensions was determined by high-performance liquid chromatography (HPLC). The HPLC analysis was performed on a Waters HPLC system consisting of a Waters 1525 binary pump, a Waters 2487 dual λ absorbance detector, and a Waters 717 plus autosampler and equipped with a Waters C18 column (5 μm, 0.46 × 25 cm). The mobile phase was composed of acetonitrile and water (40:60, v/v). The flow rate was 1.0 mL/min, and the detection wavelength was 240 nm [28]. The EE was calculated as follows.

$$\mathrm{EE \%}=\frac{{{\text{W}}}_{\mathrm{the\,amount\, of\, drug\, in \,NEs\, suspensions}}- {{\text{W}}}_{\mathrm{the\, amount\, of\, drug\, in \,filtrate}}}{{{\text{W}}}_{\mathrm{the\, amount\, of \,drug\, in\, NEs\, suspensions}}}\times 100\mathrm{\%}$$

##### Differential scanning calorimetry (DSC) and fourier transform infrared spectroscopy (FTIR)

The physical state of DEX in different formulations was analyzed by differential scanning calorimeter (Netzsch, Germany). After freeze-drying, the samples were weighed and scanned (50–300 °C) at a rate of 10 °C/min under nitrogen flow. The intermolecular interaction was analyzed using FTIR (Niclet is50, Thermo, USA). The spectrum was obtained using the KBr disk method in the range of 500 cm^−1^ to 4000 cm^−1^.

##### In vitro release

The in vitro DEX release behavior from DEX solution, NEs, and SC-NEs containing different amounts of SC (5, 10, 20, 40, 80%) was investigated by a dialysis method. Briefly, 0.5 mL of SC-NEs was dropped into a dialysis bag (MWCO: 8 kDa) and placed in 10 mL of simulated tear fluid at 35 ± 0.5 °C with continuous shaking (120 rpm). At the designated time point, an aliquot of the sample was taken out and replaced with a fresh medium. The released DEX content was determined by the HPLC method as described above.

##### In vitro stability

The in vitro stability of different formulations was studied at 4 °C. The drug content changes were determined at predetermined time points.

### Expression of OCTN2 and ATB0, + in human corneal epithelial cells and corneal tissues

#### Expression of OCTN2 and ATB.^0,+^ in human corneal epithelial cells (HCECs)

The expression of OCTN2 and ATB^0,+^ in HCECs was observed by immunofluorescence. HCECs were seeded on a 12-mm coverslip in 24-well plat at 1 × 10^5^ cells/well density. After cells grew to 90% confluence, the cells were fixed with 4% paraformaldehyde for 20 min and blocked with 3% BSA solutions for 30 min. Then, the primary antibody against ATB^0,+^ and OCTN2 was added to the cells and cultured for 12 h at 4 °C. Alexa Fluor 594 labeled secondary antibody was added to the cells and incubated for 60 min at 37 °C. The cell nucleus was stained with DAPI. Lastly, the cells were observed by fluorescence microscope (Carl Zeiss, Germany).

#### Expression of ATB^0,+^ and OCTN2 in rabbit cornea

The expression of ATB^0,+^ and OCTN2 in rabbit corneal tissue was also examined by immunofluorescence analysis. The rabbits were euthanized with air injection. The corneal tissues were excised and rinsed with ice-cold pH 7.4 phosphate-buffered saline. For immunofluorescence analysis, the corneal tissues were fixed with 4% paraformaldehyde and paraffin sections of 10 μm were prepared. The sections were blocked with 3% BSA solutions for 1 h. The samples were stained with rabbit anti-ATB^0,+^ antibody and rabbit anti-OCTN2 antibody for 12 h. Lastly, the corneal tissues were observed by fluorescence microscope (Carl Zeiss, Germany).

### Cellular uptake and mechanism studies in HCECs

#### Cellular uptake of SC-NEs

HCECs were seeded in a 24-well plate at 1 × 10^5^ cells/well density and cultured for 24 h. Fluorescence dye coumarin 6 (C6)-loaded NEs or SC-NEs were prepared as the same as the procedure in "Preparation and characterization of stearoyl L-carnitine-modified nanoemulsions (SC-NEs)" section except that DEX was replaced with C6. When the cells reach 90% confluence, C6 (2.5 μg/mL)-loaded NEs (C6 NEs) or SC-NEs (C6 SC-NEs) in NaCl buffer (140 mM NaCl, 25 mM Hepes/Tris, 5.4 mM KCl, 1.8 mM CaCl_2_, 0.8 mM MgSO_4_, and 5 mM glucose, pH 7.4) was added to the cells and cultured for 1 h. Next, the cells were rinsed with cold NaCl buffer 3 times and were solubilized by RAPI buffer for 60 min. The fluorescence intensity was measured using a multimode microplate reader (Bio Tek, USA) at λ_ex_ = 460 nm and λ_em_ = 505 nm. Protein concentration was measured by a BCA kit.

For the fluorescence imaging study, HCECs were seeded on a 12-mm coverslip in a 24-well plate at 1 × 10^5^ cells/well density. After cells reach 90% confluence, the medium was removed, and C6 SC-NEs in NaCl buffer were added and cultured for 1 h. The nucleus was stained by DAPI. The cell was observed under a Leica fluorescence microscope.

#### Cellular uptake mechanisms

To confirm the effect of ATB^0,+^ and OCTN2 in the uptake of SC-NEs, α-methyl-DL-tryptophan (α-MT, 2.5 mM) was selected as a specific inhibitor of ATB^0,+^, and L-carnitine (10 mM) was selected as a competitive inhibitor for both ATB^0,+^ and OCTN2.

To investigate Na^+^ and Cl^−^ on the cellular uptake of SC-NEs, Na^+^ or Cl^−^ free buffer was selected in this study. Na^+^ free buffer was prepared by substituting NaCl in the buffer with methylglucamine hydrochloride. The gluconic acid sodium salt, gluconic acid potassium salt, and gluconic acid calcium salt were used instead of NaCl, KCl, and CaCl_2_ for preparing Cl^−^ free buffer.

To investigate the endocytosis pathway of SC-NEs, different endocytosis inhibitors (chlorpromazine: 50 μM, indomethacin: 100 μM, colchicine: 10 μM, quercetin: 10 μM) were added to the 24-well plate containing HCECs at 1 × 10^5^ cells/well density. After treatment for 30 min, the medium was withdrawn and C6 SC-NEs were added to cells and cultured for 1 h. Cells without treatment were set as a control group.

### Corneal permeation study

The in vivo corneal permeation of SC-NEs in rabbit eyes was evaluated by the fluorescence imaging technique. Briefly, C6 NEs and C6 10% SC-NEs (50 μL, 0.06 mg/mL) were instilled into the right eyes every 2 min for 5 times consecutively. At the predetermined time points (15 min and 60 min), the rabbits were euthanatized, and their corneas were removed for the frozen section. The cell nucleus was stained with DAPI, and the slide was observed by a Leica fluorescence microscope. The relative fluorescence intensity was quantified by Image J software.

The ex vivo corneal permeation of SC-NEs was studied using a vertical franz cell system (RYG-6B, Shanghai, China). Briefly, after New Zealand albino rabbits were euthanized, the corneas were isolated and washed with glutathione bicarbonate ringer's (GBR) solution. Then, the cornea was clipped in the middle of the donor chamber and the receptor chamber with the epithelial surface toward the donor chamber. 7 mL of glutathione bicarbonate GBR solution was added into the receptor chamber. 1 mL of DEX solution, DEX NEs and DEX 10% SC-NEs (0.1 mg/mL) was added into the donor chamber. The cell diameter is 1 cm. The water bath temperature was set at 35 ± 0.5 °C. At different time points, equal volume (20 μL) of sample was taken from the receptor chamber. The samples were diluted with methanol and drug concentration was measured by HPLC. The cumulative permeation (Q), permeability coefficient (P_app_) and steady-state flux (J_ss_) were calculated using the following equations:

$${Q}_{n}=\frac{{V}_{0}}{A}({C}_{n}+\frac{V}{{V}_{0}}\sum_{i=1}^{n-1}{C}_{i})$$

$$P_{app} = \frac{\Delta Q}{{\Delta t \cdot C_0 }}$$

$${J}_{ss}={C}_{0}\cdot {P}_{app}$$

where V_0_ is the volume of receptor chamber; V is the sampling volume; C_n_ is the drug concentration at the sampling time points; C_i_ is drug concentration at the previous time points; A is the effective diffusion area; C_0_ is the initial drug concentration;ΔQ/Δt is the slope of regression equation.

### Ocular surface retention study

Fluorescence imaging was used to investigate the ocular surface retention of SC-NEs and Rhodamine B (RhB) was selected as a fluorescence probe [29]. RhB-loaded NEs (RhB NEs) or SC-NEs (RhB SC-NEs) were prepared as the same as the procedure in "Preparation and characterization of stearoyl L-carnitine-modified nanoemulsions (SC-NEs)" section except that DEX was replaced with RhB. Rabbits were topically administered with RhB NEs and RhB 10% SC-NEs (20 μL, 1 mg/mL). The Ocular surface fluorescence signals were captured by an in vivo fluorescence imaging system (Bruker, USA) at 1, 30, and 60 min, respectively.

### In vivo pharmacokinetics

Rabbits were randomly divided into three groups and each group had three rabbits (n = 3). After topical administration with DEX solution, DEX NEs, and DEX 10% SC-NEs (50 μL, 0.5 mg/mL) into the lower conjunctival sac, the rabbits were euthanized with air injection at 10, 20, 40, 60, 90, and 120 min. After euthanized, an insulin needle was used to puncture the anterior chamber to collect aqueous humor and isolate the cornea tissue from the eyeball at each time point. The aqueous humor (0.15 mL) was mixed with 0.15 mL methanol via the vortex. The cornea tissues were homogenized with 0.5 mL methanol. Then, the aqueous humor samples and cornea homogenates were centrifuged (12,000 rpm, 10 min), and the DEX concentration in the supernatant was determined by the HPLC method as described above.

### In vivo anti-inflammatory efficacy

The in vivo anti-inflammatory efficacy of DEX SC-NEs was investigated using the EIU model in rabbits. After establishing the EIU model by intravitreal injection of Lipopolysaccharide (0.1 μgE. O111:B4, Sigma-Aldrich), DEX solution, DEX NEs, and DEX 10% SC-NEs (50 μL, 0.5 mg/mL) were dripped into the inferior conjunctival sac (4 times over 24 h). The clinical signs were evaluated with a slip lamp examination. At 24 h after uveitis induction, the rabbits were killed and the iris-ciliary body was collected for mRNA measurement. Total RNA was isolated with TRIzol reagent (Invitrogen, CA), and reverse-transcribed to cDNA using HiScript® III RT SuperMix for qPCR (+ gDNA wiper) kit (Vazyme, China). The qRT-PCR was achieved by ChamQ Universal SYBR qPCR Master Mix (Vazyme, China). The primer sequences of monocyte chemoattractant protein 1 (MCP-1), Matrix metalloproteinases 1 (MMP-1), and vascular cell adhesion molecule 1(VCAM-1) are listed in Additional file 1: Table S3. GAPDH was used as a reference gene. In addition, the eyeballs were fixed in formaldehyde, embedded in paraffin, sectioned, and stained with hematoxylin and eosin (H&E) for histopathological examination.

### Ocular safety assessment

#### In vitro cytotoxicity test in HCECs.

The cytotoxicity assay was performed on HCECs. Briefly, HECEs (1 × 10^4^ cells/well) were seeded into the wells of 96-well plates and incubated overnight. Then, DEX 10% SC-NEs were added to the wells. After 24 h incubation, CCK8 reagent was added to the wells and cultured for 1 h. The absorbance values were determined with a microplate reader (Bio Tek, Vermont, USA) at 490 nm.

#### In vivo ocular irritation in rabbit eyes

A modified Draize eye irritation test was used to investigate the ocular safety of SC-NEs in vivo [30]. First, the rabbit eyes were topically administered with DEX solution, DEX NEs, and DEX 10% SC-NEs (50 μL, 0.5 mg/mL). The clinic signs of the ocular anterior segment were assessed by slit lamp at 24 h after instillation. The corneal surface integrity was also evaluated by fluorescein staining. Finally, the rabbit was euthanatized by intravenous air injection, and the eyeball was taken out to prepare hematoxylin and eosin (H&E) pathological sections.

#### Statistical analysis

All values are presented as mean ± standard deviation (SD). The Student’s t-test was used to analyze the statistical difference between the data. The P-value < 0.05 was considered statistically significant.

## Results and discussion

### Synthesis and characterization of stearoyl L-carnitine

L-carnitine is a zwitterion, which contains three polar moieties, a trimethyl-amino moiety, a carboxyl moiety, and a hydroxyl moiety. Among them, the trimethyl-amino moiety and carboxyl moiety are indispensable for recognition by the transporter [31]. Moreover, L-carnitine is a small hydrophilic molecule, which could not be adsorbed on the NEs surface. Therefore, the hydroxyl moiety of L-carnitine was selected to react with the carboxyl moiety of stearic acid to form an ester of stearoyl L-carnitine.

The alkyl chain of stearoyl L-carnitine can insert into the lipid core of NEs by hydrophobic-hydrophobic interactions. The synthetic process was described in the supplementary information. The chemical structure of stearoyl L-carnitine was confirmed by proton nuclear magnetic resonance (^1^H-NMR) (Additional file 1: Fig. S1) and mass spectrum (MS) (Additional file 1: Fig. S2), which demonstrated the successful synthesis of stearoyl L-carnitine.

### Preparation and characterization of SC-NEs

NEs are composed of an oil phase, an aqueous phase, and a surfactant. Oil is one of the most important excipient in the NEs formulation. The oil phase with high solubility of drugs is generally used to develop NEs. The solubility of DEX in different oils, including medium-chain triglycerides, soybean oil, olive oil, and Labrafil® M1944 CS was screened (Additional file 1: Fig. S3), the highest drug solubility was found in Labrifil® M 1944 CS. Therefore, Labrafil® M1944 CS was selected as the oil phase of the NEs to improve the solubility of DEX. Next, the DEX NEs were prepared by emulsion solvent evaporation method. Tween 80 was used as the surfactant, and the effect of the amount of oil phase on the physicochemical properties of the DEX NEs was screened (Additional file 1: Table S1). When the amount of Labrifil® M 1944 CS was set at 50 mg, DEX NEs had higher drug content and EE, and this formulation was selected to further prepare DEX SC-NEs.

**Fig. 1:**
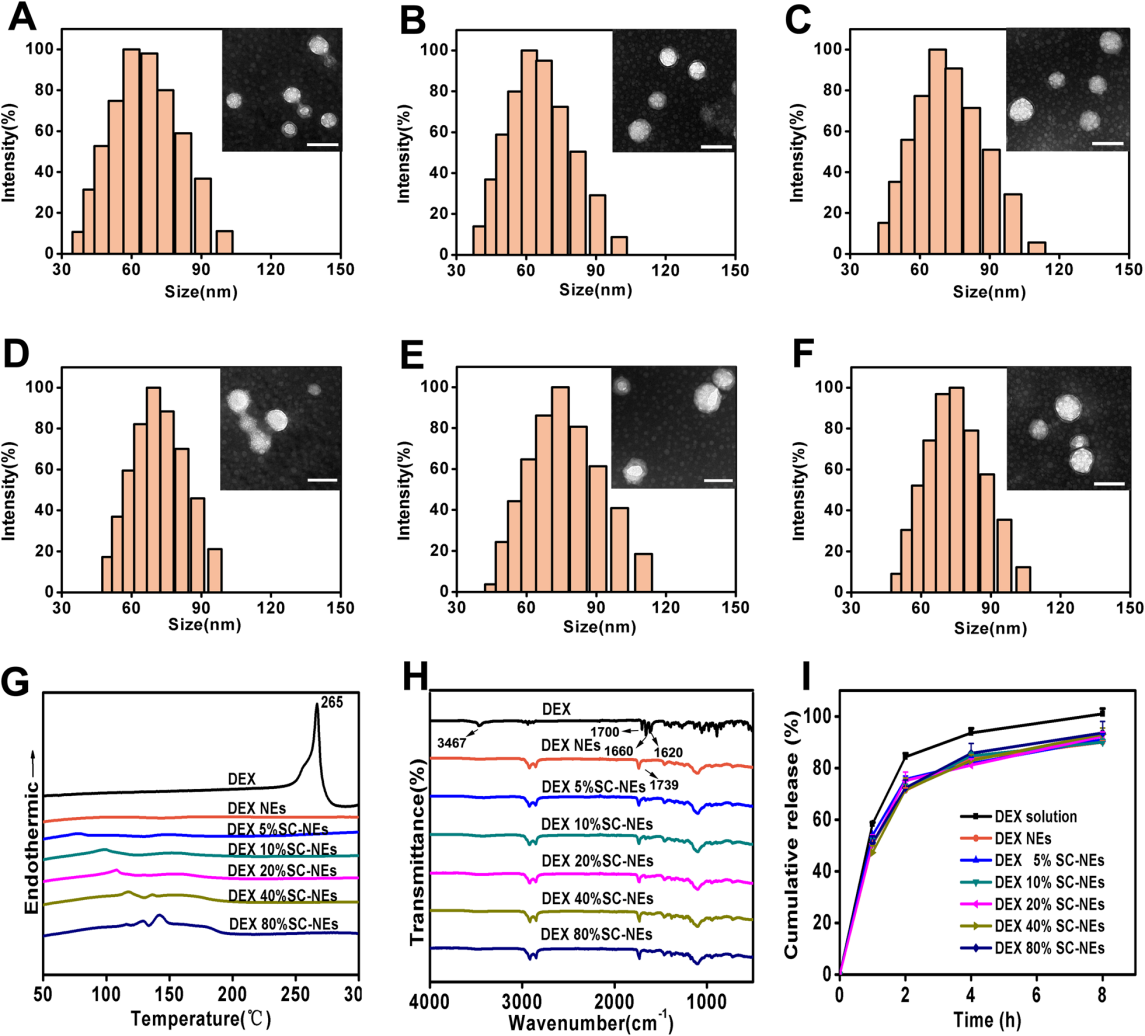
Physicochemical characterization of stearoyl L-carnitine (SC)-modified nanoemulsions (SC-NEs) with varying SC modification ratios. Particle size distribution and TEM images of DEX NEs (**A**), DEX 5% SC-NEs (**B**), DEX 10% SC-NEs (**C**), DEX 20% SC-NEs (**D**), DEX 40% SC-NEs (**E**), DEX 80% SC-NEs (**F**) (scale bar in TEM images = 100 nm); DCS (**G**), FTIR (**H**), and in vitro drug release profiles (**I**) of DEX NEs modified with different ratios of SC (n = 3)

The DEX SC-NEs were characterized in terms of size, morphology, zeta potential, EE, DSC, FTIR and in vitro drug release. As shown in Fig. 1A–F and Additional file 1: Table S2, all SC-NEs exhibited small particle size, spherical morphology, negative zeta potential and high encapsulation efficiency (> 90%). The physical state of DEX in the NEs was analyzed by DSC (Fig. 1G). The DSC curve of DEX showed a sharp endothermic peak at 266 °C. However, this peak disappeared in the curve of the various NEs formulations, indicating the drug was in an amorphous sate in the NEs formulations. The possible interactions between DEX and the matrix were analyzed by FTIR. As shown in Fig. 1H, the absorption band at 3467 cm^−1^ was attributed to the stretching vibration of -OH bond; the stretching vibrations at 1620, 1660 and 1700 cm^−1^ were attributed to C = O bonds [32]. However, the absorption band at 3467 cm^−1^ almost disappeared, and the absorption bands at the range of 1620–1700 cm^−1^ were shifted to 1739 cm^−1^, indicating the presence of hydrogen bond interaction between DEX and the lipid matrix [33].

The in vitro drug release profiles of various NEs formulations are shown in Fig. 1I. All the NEs formulations exhibited sustained-release profiles compared with that of free DEX solution. There was no significant difference in drug release profiles among the different NEs formulations, indicating that the SC modification ratio did not influence the drug release profiles of NEs. In addition, stability experiments were performed at 4 °C (Additional file 1: Fig. S4). The drug content of DEX NEs, DEX 5% SC-NEs, DEX 10% SC-NEs and DEX 20% SC-NEs could keep over 90% after 2 days. However, the drug content of DEX 40% SC-NEs and DEX 80% SC-NEs decreased by 26% and 18%, respectively. The results indicated that excessive SC content is not conducive to the stability of the NEs.

### Expression of OCTN2 and ATB^0,+^ in HECEs and rabbit cornea

**Fig. 2:**
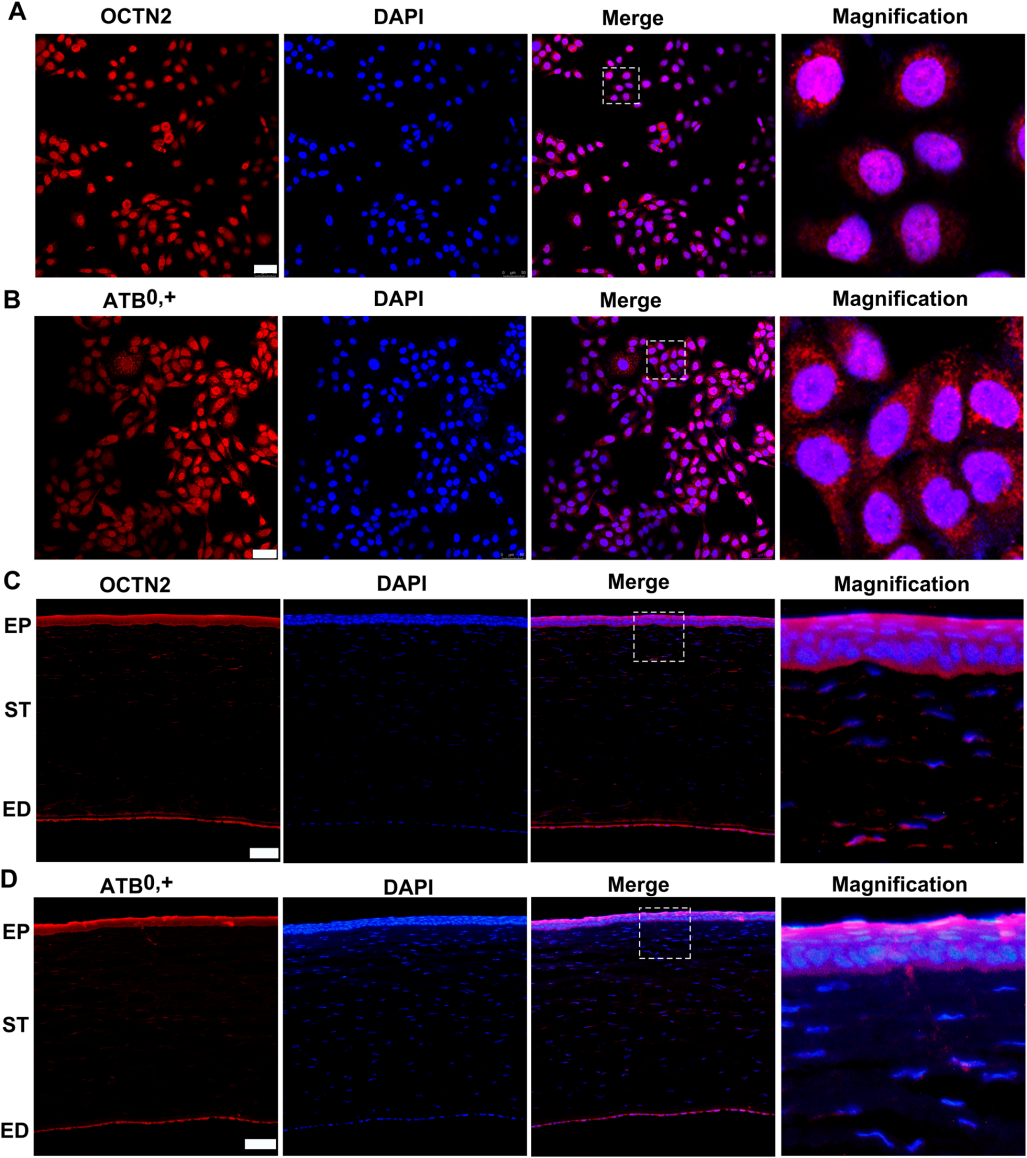
Immunofluorescence images of OCTN2 and ATB^0,+^ in HECEs (**A**, **B**, scale bar = 50 μm) and rabbit cornea tissue (**C**, **D**, scale bar = 30 μm, EP: Epithelium, ST: Stroma, ED: Endothelium)

Immunofluorescence was used to analyze the expression of OCTN2 and ATB^0,+^ in HECEs and rabbit cornea tissue. As shown in Fig. 2A, B OCTN2 and ATB^0,+^ were highly expressed in HECEs. In addition, ATB^0,+^ and OCTN2 expression in rabbit cornea tissue was also determined by immunofluorescence analysis. As shown in Fig. 2C, D the cornea consists of three main layers in series: the epithelium, the stroma, and the endothelium [34, 35]. The corneal epithelium is the outermost layer of the cornea, and is composed of 4–6 layers of nonkeratinized, stratified squamous epithelial cells. The corneal stroma is highly hydrated fibrous acellular tissue. The corneal endothelium is very thin, and is composed of a single layer of polygonal cells.

ATB^0,+^ and OCTN2 were mainly distributed on the corneal epithelium and endothelium, and were rarely distributed in the corneal stroma. These results were consistent with the earlier reported studies [36].

### Cellular uptake and mechanisms of SC-NEs in HCECs

**Fig. 3:**
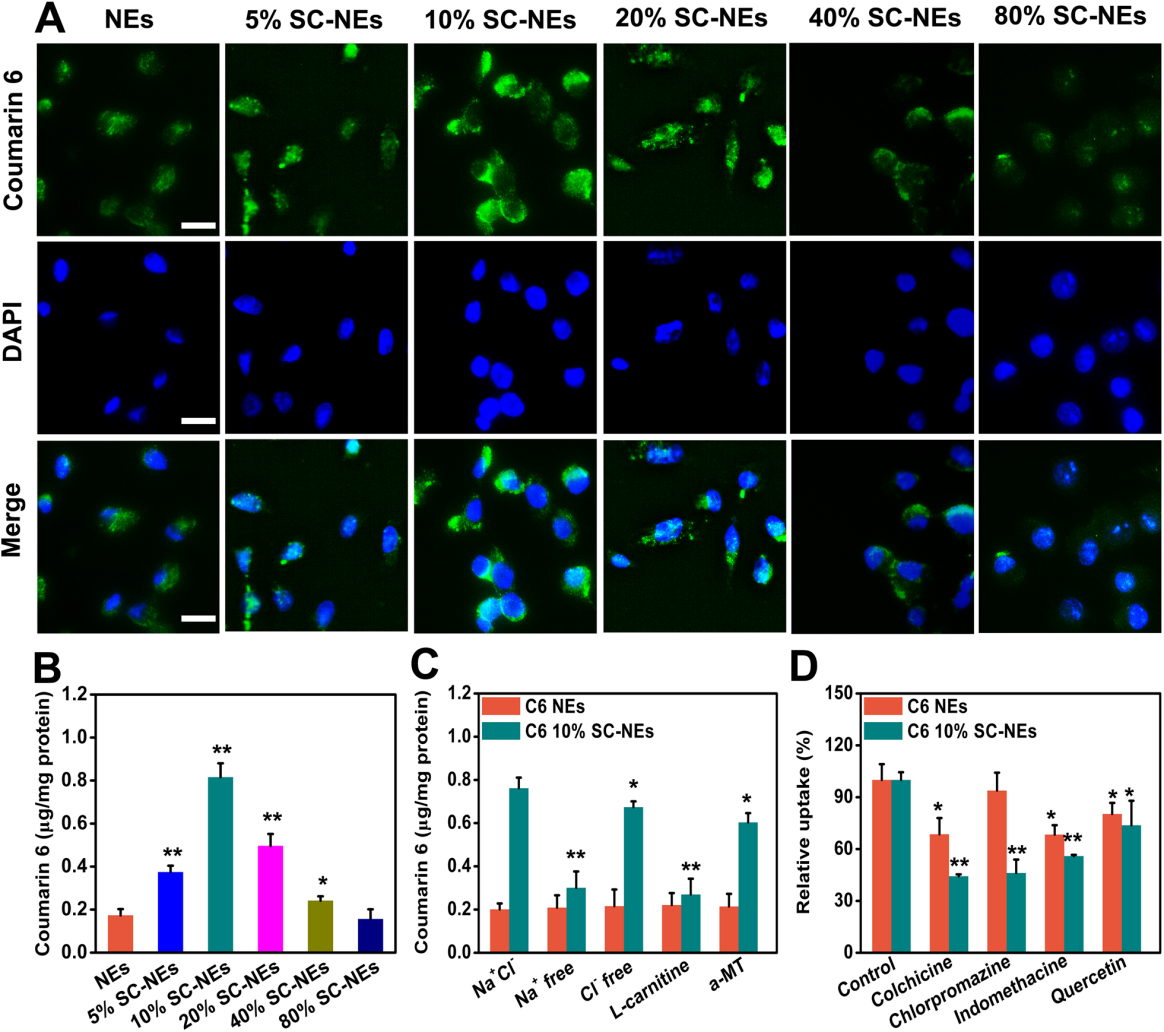
Cellular uptake and mechanisms of SC-NEs in HCECs. **A** Cellular uptake of SC-NEs with varying SC modification ratios observed by fluorescence microscope (scale bar = 20 μm); **B** Quantitative cellular uptake of SC-NEs with varying SC modification ratios (n = 3, *P < 0.05, **P < 0.01, compared with NEs); **C** Quantitative cellular uptake of NEs and 10% SC-NEs in different buffers: NaCl buffer, Na^+^ free buffer, Cl^−^ free buffer, NaCl buffer with L-carnitine, and NaCl buffer with α-methyl-DL-tryptophan (α-MT) (n = 3, *P < 0.05, **P < 0.01, compared with 10% SC-NEs in NaCl buffer); **D** Influence of endocytosis inhibitors on the cellular uptake of NEs and 10% SC-NEs (n = 3, *P < 0.05, **P < 0.01, compared with Control)

The corneal epithelium is a crucial barrier that limits the drug penetrating intraocular tissues. Here, we used HECEs to explore the cellular uptake of NEs with various SC modification ratios by fluorescence imaging analysis. As shown in Fig. 3A, the green fluorescence of NEs increased first and then gradually decreased with the increase in the SC ratio, and the strongest fluorescence signal was produced at the SC content of 10% in the NEs. Besides, the quantitative analysis was performed and the result was shown in Fig. 3B, it was discovered that 10% SC-NEs exhibited the strongest fluorescence intensity among all SC-NEs formulations, which was 4.72-fold stronger than that of unmodified NEs. These results indicated that the SC-NEs with appropriate ligand density could achieve optimal cellular uptake, and neither too low nor too high a ratio of SC modification was not beneficial for the cellular uptake of SC-NEs. Therefore, 10% SC-NEs were selected for the following experiments.

It was reported that Na^+^ and Cl^−^ could affect the cellular uptake of L-carnitine-modified nanoparticles by regulating the state of transporters [25, 37]. That is, L-carnitine-modified nanoparticles and Na^+^/Cl^−^ bind to the specific sites of OCTN2 or ATB^0,+^, and the transporters change their conformation from outward-facing to a stable occluded state, triggering membrane invagination and endocytosis process. Without Na^+^/Cl^−^, L-carnitine-modified nanoparticles could bind to transporters but easily detach from the transporters. Therefore, we evaluated the effect of Na^+^ and Cl^−^ on the cellular uptake of SC-NEs. As shown in Fig. 3C, the cellular uptake of unmodified NEs was not influenced by the Na^+^ and Cl^−^ in the medium. On the contrary, both Na^+^ and Cl^−^ obviously influenced the cellular uptake of SC-NEs, and the effect of Na^+^ was greater than that of Cl^−^. More specifically, the absence of Na^+^ in the culture medium reduced the cellular uptake of 10% SC-NEs by 2.53-fold, whereas the absence of Cl^−^ in the culture medium just decreased the cellular uptake of 10% SC-NEs by 0.88-fold. These phenomena were attributed to the different transport characteristics of OCTN2 and ATB^0,+^. ATB^0,+^ is a Na^+^- and Cl^−^-coupled low-affinity transporter for L-carnitine, while OCTN2 is a Na^+^-dependent high-affinity L-carnitine transporter [37]. To discern the involvement of OCTN2 and ATB^0,+^ in the cellular uptake of SC-NEs, α-methyl-DL-tryptophan (α-MT) was used as a specific inhibitor for ATB^0,+^, and L-carnitine was used as the inhibitor for both OCTN2 and ATB^0,+^ [20]. It was discovered that both inhibitors decreased the cellular uptake of SC-NEs, which suggested that both OCTN2 and ATB^0,+^ participated in the cellular uptake of SC-NEs.

We further used different endocytosis inhibitors to track the potential pathways of cellular uptake of SC-NEs (Fig. 3D). The presence of colchicine, chlorpromazine, and indomethacin significantly reduced the cellular uptake of SC-NEs, suggesting that micropinocytosis-, clathrin- and caveolae-mediated endocytosis pathways were involved in the uptake process. In addition, the presence of colchicine and chlorpromazine decreased the cellular uptake of NEs, suggesting that the cellular uptake of unmodified NEs was mainly dependent on macropinocytosis and clathrin-mediated pathways. Both unmodified NEs and SC-NEs displayed lower cellular uptake in the presence of quercetin, indicating that caveolin and clathrin-independent endocytosis pathway was also involved in the uptake process, but the effect of quercetin was relatively weaker compared with the other endocytosis inhibitors. The difference in the endocytosis pathway between unmodified NEs and SC-NEs may be attributed to the altered surface property of nanoemulsions, namely, L-carnitine modification [38].

**Fig. 4:**
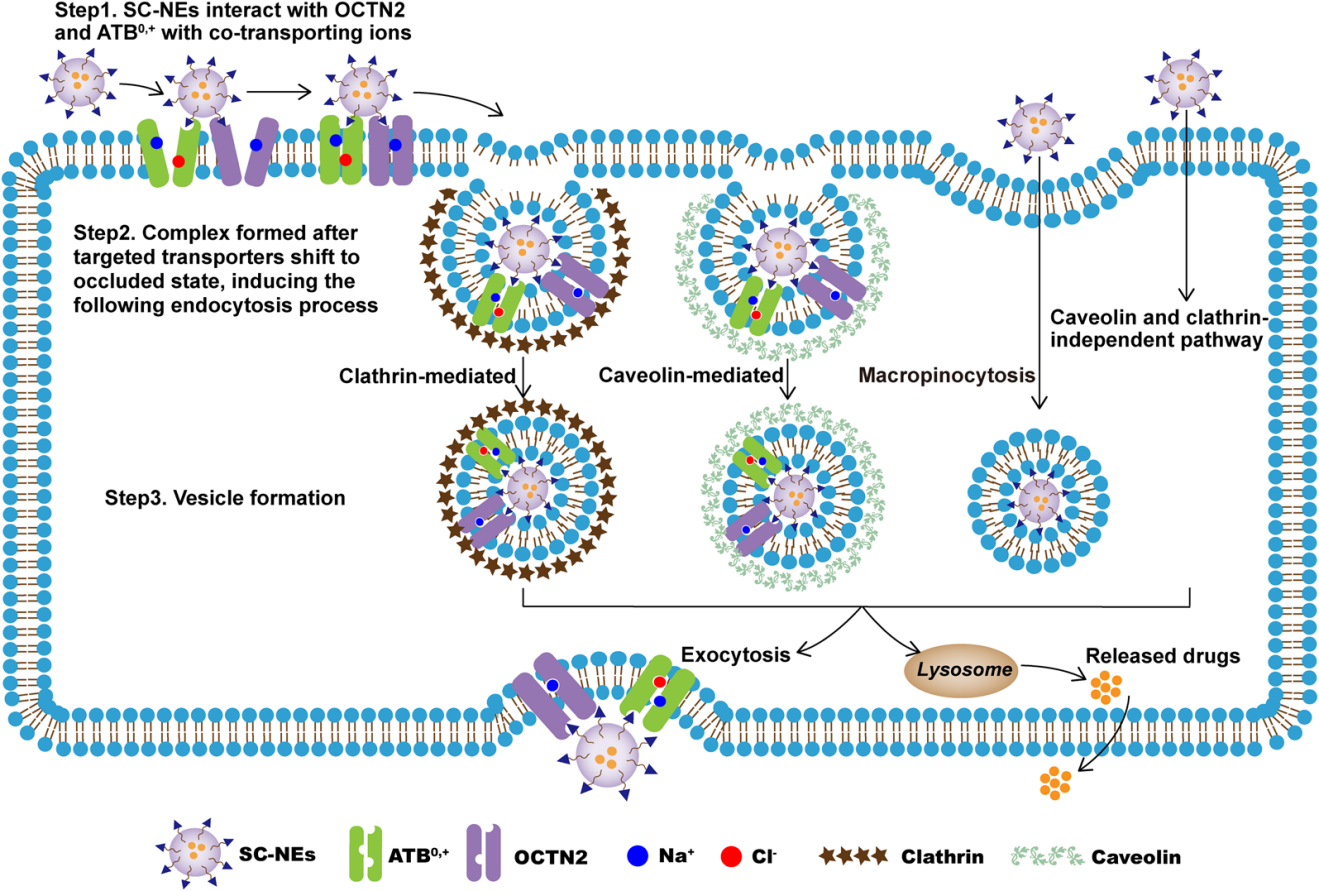
Schematic illustration of proposed transport mechanisms for SC-NEs in HCECs. SC-NEs and Na^+^/Cl^−^ bind to the specific sites of OCTN2 and ATB^0,+^, and the transporters change their conformation from outward-facing to a stable occluded state, triggering membrane invagination and clathrin- and caveolin-medicated endocytosis process. In addition, macropinocytosis and caveolin and clathrin-independent pathway are also involved in the cellular uptake and transport of SC-NEs

Based on the above results, the transport mechanisms of SC-NEs in the corneal epithelium are depicted in Fig. 4. Initially, SC-NEs interact with both OCTN2 and ATB^0,+^ in the presence of Na^+^ and Cl^−^. The transporters change their conformation from outward-facing to occluded state, resulting in the formation of a stable complex (SC-NEs-OCTN2-ATB^0,+^-Na^+^-Cl^−^), which triggers membrane invagination and clathrin- and caveolin-medicated endocytosis. Then, the lipid vesicle containing SC-NEs-OCTN2-ATB^0,+^-Na^+^-Cl^−^ complex would exit from the cell by exocytosis. Apart from exocytosis, a portion of the lipid vesicle-containing complex would fuse with the lysosome. The complex would be degraded and the released drugs can diffuse freely across the membrane. Macropinocytosis, a nonspecific endocytosis process, is also involved in the cellular uptake and transport of SC-NEs. In addition, only very few SC-NEs are internalized by caveolin and clathrin-independent pathway.

Generally, the corneal epithelium is the main barrier limiting drug entry into the eye, while the stroma and endothelium have very little resistance to transcorneal penetration [39, 40]. After the permeation across the corneal epithelium, SC-NEs would diffuse through the stroma, and then transport across the corneal endothelium through transporter-mediated transcytosis. The transcytosis pathway may be similar to the transport of SC-NEs in the corneal epithelium. In summary, SC-NEs could enhance the cellular uptake and transport in the corneal tissues, facilitating the improvement of corneal permeability and ocular drug bioavailability.

### Corneal permeation study of SC-NEs

**Fig. 5:**
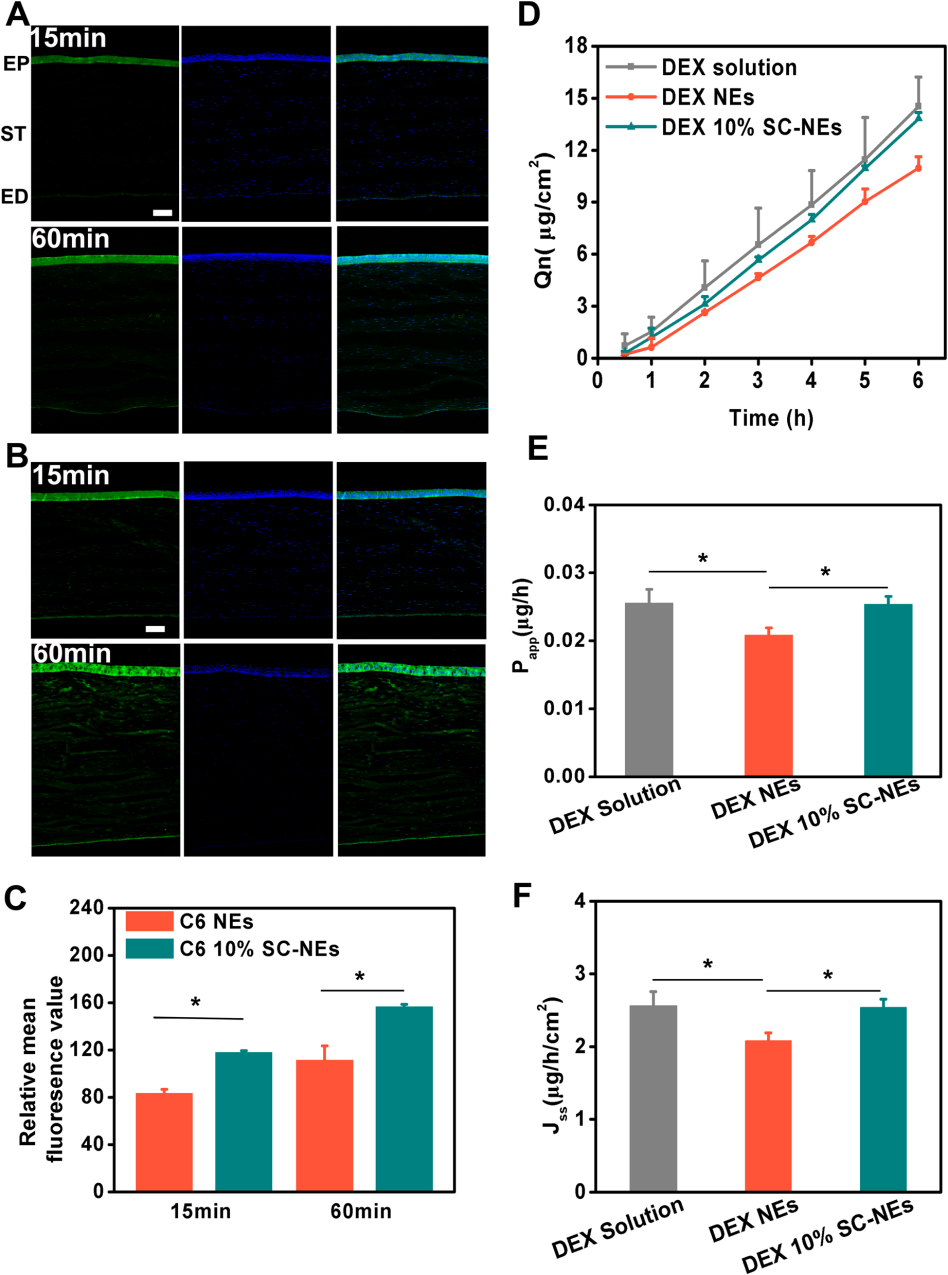
Corneal permeation study of SC-NEs in rabbit eyes. Fluorescence images of corneal tissues at 15 min and 60 min after topical instillation of C6 NEs (**A**) (EP: Epithelium; ST: Stroma; ED: Endothelium; scale bar = 5 μm) and C6 10% SC-NEs (**B**); Relative fluorescence intensity of corneal tissues at 15 min and 60 min after topical instillation (**C**); Transcorneal cumulative permeation profiles (**D**), permeability coefficient (P_app_) (**E**) and steady-state flux (J_ss_) (**F**) from the transcorneal permeation study of DEX solution, DEX NEs and DEX 10% SC-NEs (n = 3).*P < 0.05

The corneal epithelium is considered to be the main barrier to intraocular drug permeation through topical administration. Thus, trans-corneal permeation is the predominant way for drug transport into intraocular tissues. To observe the permeability of unmodified NEs and 10% SC-NEs, a fluorescence imaging technique was adopted to trace the corneal fluorescence distribution. Figure 5A, B show obvious heterogeneity distribution of the fluorescence signal in the cornea. The fluorescence signal was mainly distributed in the corneal epithelium and was relatively weaker in the corneal stroma and endothelium. This phenomenon verified that the corneal epithelium acted as the crucial physiological barrier function in the cornea. For both NEs and SC-NEs, the fluorescence signal in the entire corneal tissue (epithelium, stroma, and endothelium) was enhanced from 15 to 60 min, indicating that the corneal permeation of NEs was time-dependent. Most importantly, the fluorescence signal of 10% SC-NEs in the corneal tissue was stronger than that of unmodified NEs at 15 min and 60 min after instillation, indicating the surface of nanoemulsions modification with SC could increase corneal permeation and distribution. This result was attributed to the transporter-mediated active transport by L-carnitine-OCTN2 (and ATB^0,+^) interaction. Furthermore, the fluorescence intensity was quantitatively analyzed and the result was shown in Fig. 5C. The relative fluorescence intensity of 10% SC-NEs was significantly higher than that of NEs at 15 min and 60 min. The ex vivo transcorneal permeation of DEX was evaluated using a Franz diffusion cell. As show in Fig. 5D–F, compare to DEX NEs, the values of J_ss_ from DEX 10% SC-NEs were significantly increased by 1.2-fold. The result indicated that SC modification could increase the transcorneal permeation of NEs. However, the values of J_ss_ from DEX NEs were lower than that of DEX solution. The decreased permeability of DEX NEs could be due to the sustained release as compared to DEX solution [41]. In summary, SC-NEs could increase corneal permeation, which was beneficial for the improvement of ocular drug bioavailability.

### Ocular surface retention study of SC-NEs

**Fig. 6:**
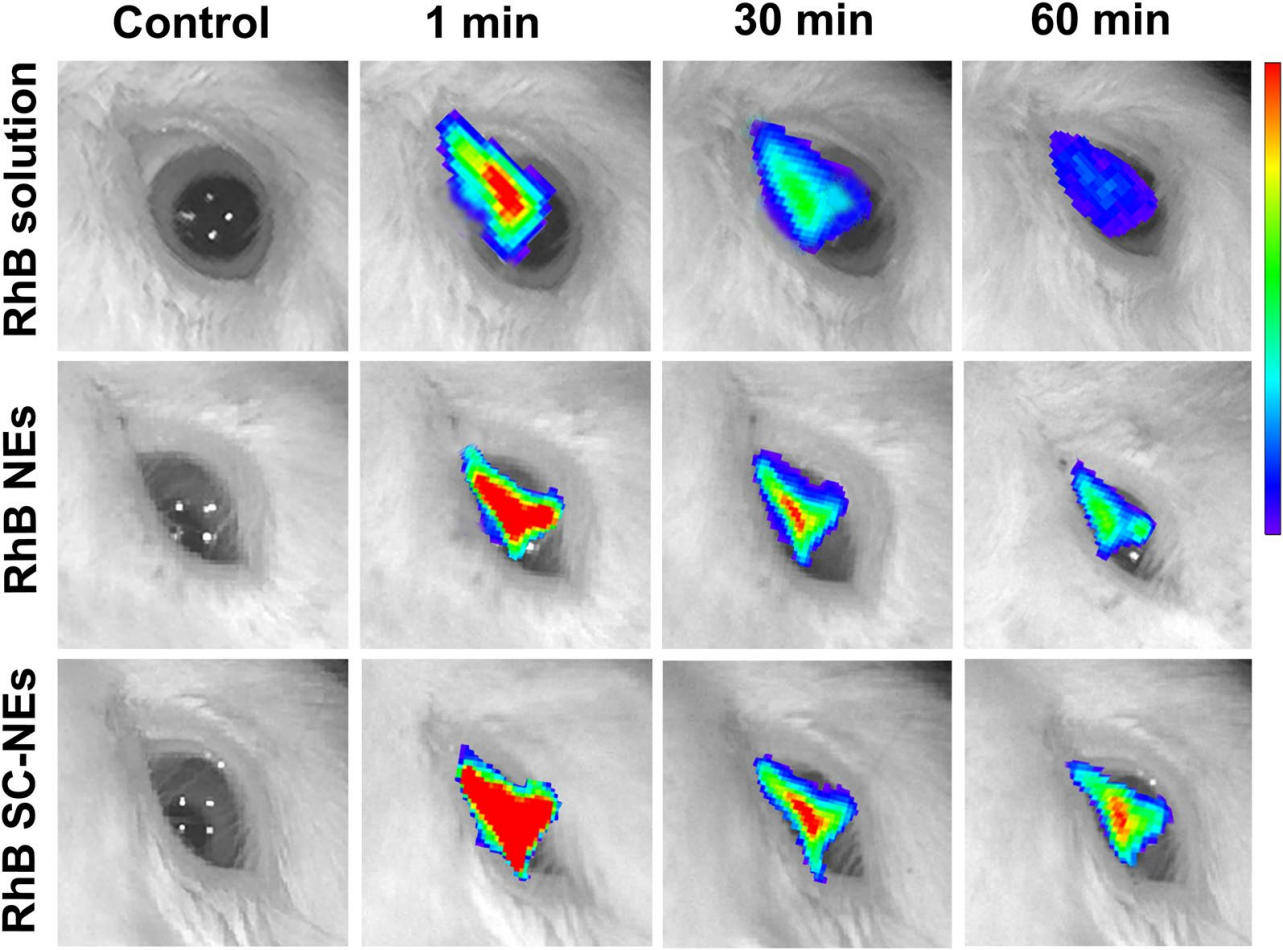
Ocular surface retention study of SC-NEs in rabbit eyes. Fluorescence images of rabbit eyes after topical instillation of RhB solution, RhB NEs, and RhB 10% SC-NEs at different time points

To visualize the ocular surface retention ability of NEs and SC-NEs, rabbit eyes were topically instilled with RhB NEs and RhB SC-NEs for fluorescence imaging analysis. As shown in Fig. 6, the fluorescence signal gradually decreased with the time increased from 1 to 60 min for all the formulations. RhB solution showed an extremely weak fluorescence signal at 60 min after instillation, indicating the RhB solution could be rapidly cleared from the ocular surface owing to blinking and lacrimal drainage. RhB NEs showed a stronger fluorescence signal than that of RhB solution at every time point, suggesting NEs could improve the ocular surface retention time of RhB. Recent studies have demonstrated that lipid components in the nanoemulsions could interact with the lipid layer of the tear film, facilitating the prolongation of the ocular surface retention time; besides, surfactant in the nanoemulsions could reduce the surface tension between the nanoemulsion eye drops and cornea through improving the spreading coefficient, which was also beneficial for the extension of ocular residence time [14]. Additionally, RhB SC-NEs exhibited the strongest fluorescence signal compared with RhB NEs and RhB solution at any test time point. This result was ascribed to the intrinsic properties of nanoemulsions and the ligand-receptor interaction between L-carnitine on the surface of SC-NEs and OCTN2/ATB^0,+^ on the corneal epithelium which further prolonged the ocular surface retention time of nanoemulsions.

### In vivo pharmacokinetics of SC-NEs

**Fig. 7:**
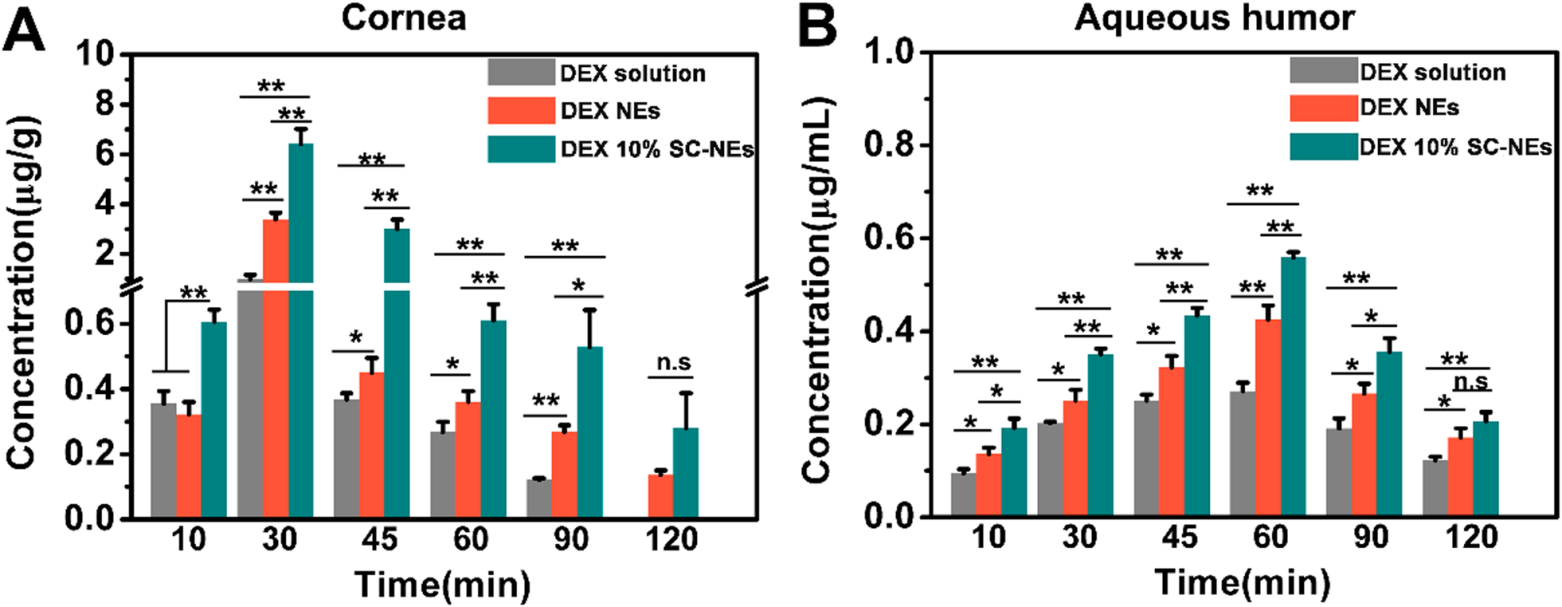
In vivo pharmacokinetic behavior of DEX 10% SC-NEs in rabbit eyes. Drug concentrations in the cornea (**A**) and aqueous humor (**B**) after topical instillation of DEX solution, DEX NEs, and DEX 10% SC-NEs in the rabbit at different time points (n = 3). *P < 0.05, **P < 0.01. No significant difference was marked as n.s

To further confirm that SC-NEs can improve precorneal drug retention and corneal permeation and finally lead to an increase in ocular bioavailability, the in vivo pharmacokinetics of DEX SC-NEs were evaluated after a single topical administration. As shown in Fig. 7A, DEX SC-NEs achieved higher DEX concentration in the cornea than NEs at predetermined time points. Furthermore, it was evident that DEX SC-NEs obtained higher drug concentration in aqueous humor than that of DEX NEs from 10 to 90 min (Fig. 7B). Meanwhile, pharmacokinetic parameters including the area under the concentration–time curve (AUC) and maximum drug concentration (C_max_) were calculated by DAS 2.0. The AUC of DEX SC-NEs in the cornea was 204.06 ± 18.42 μg/g min, which was significantly higher than that of DEX NEs (101.35 ± 4.49 μg/g min) and DEX solution (39.70 ± 3.66 μg/g) (Table 1). The AUC of DEX SC-NEs in aqueous humor was 54.76 ± 2.95 μg/mL min, which was significantly higher than that of DEX NEs (43.10 ± 2.57 μg/mL min) and DEX solution (33.05 ± 1.87 μg/mL min) (Table 1). In addition, the C_max_ of DEX SC-NEs in both cornea and aqueous humor was greatly higher than that of DEX NEs and DEX solution (Table 1). These results further confirmed that SC-NEs could improve corneal permeation and thus increase intraocular drug bioavailability. The reasons for increased intraocular drug bioavailability are summarized as follows: (i) the lipid composition in nanoemulsions could interact with the lipid layer of the tear film, resulting in prolonged precorneal retention time [14]; (ii) L-carnitine-modified nanoemulsions could be recognized by OCTN2 and ATB^0,+^ on the corneal epithelium via ligand-transporter interaction. This interaction imparts nanoemulsions with enhanced bioadhesiveness in the cornea and induces transporter-mediated transcytosis, finally ensuring the improvement in corneal permeation; (iii) the prepared nanoemulsions with small size (about 70 nm) could distribute homogeneously on the ocular surface, facilitating deep corneal permeation [42]; (iv) Surfactants in nanoemulsions could probably open the corneal epithelial tight junction, which is beneficial to the drug permeation through paracellular route [14].

**Table 1:** Pharmacokinetic parameters of DEX solution, DEX NEs, and DEX 10% SC-NEs after topical instillation in rabbits (n = 3)

Ocular tissues	Pharmacokinetic parameter	DEX solution	DEX NEs	DEX 10% SC-NEs
Cornea	AUC_(0-∞)_ (μg/g·min)	39.70 ± 3.66	101.35 ± 4.49*	204.06 ± 18.42*^, #^
	C_max_ (μg/g)	1.00 ± 0.17	3.40 ± 0.26*	6.43 ± 0.60*^,#^
Aqueous humor	AUC_(0-∞)_ (μg/mL·min)	33.05 ± 1.87	43.10 ± 2.57*	54.76 ± 2.95*^, #^
	C_max_ (μg/mL)	0.27 ± 0.02	0.43 ± 0.03*	0.56 ± 0.01*^, #^

^*^P < 0.05, compared with DEX solution

^#^P < 0.05, compared with DEX NEs

### In vivo anti-inflammatory efficacy

**Fig. 8:**
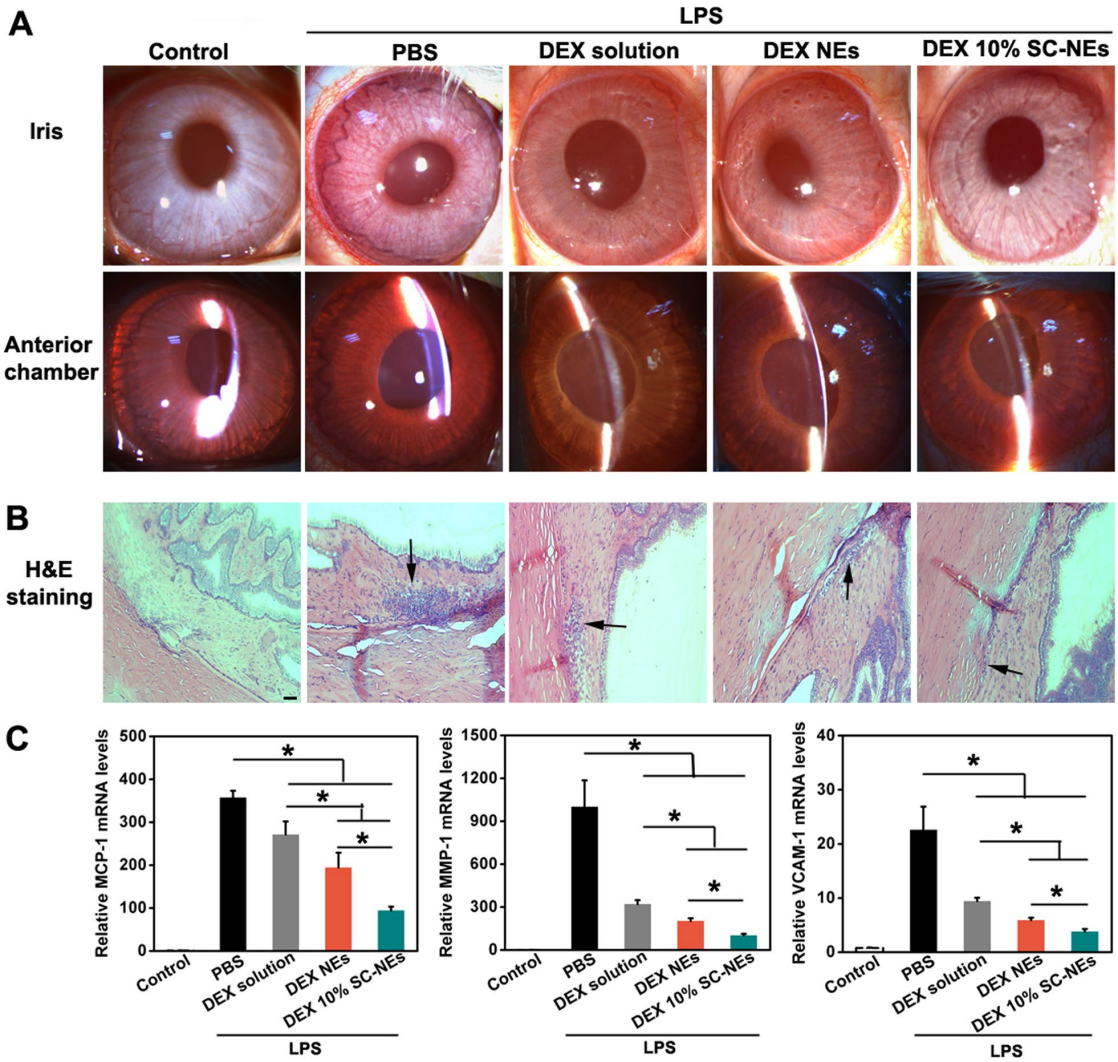
In vivo anti-inflammatory efficacy of DEX 10% SC-NEs in a rabbit model of EIU. **A** Slit-lamp observation of clinical symptoms (e.g., iris hyperaemia, exudation); (**B**) H&E sections of the iris-ciliary body from different groups and black arrow indicated inflammatory cell infiltration (bar = 50 μm); (**C**) Relative mRNA levels of MCP-1, MMP-1, and VCAM-1 in iris-ciliary body from different groups (n = 3). *P < 0.05

The rabbit model of EIU is a classic model for studying human anterior uveitis, which mainly involves inflammation of iris-ciliary body. After treatment with different groups, the clinical signs of the anterior segment were observed by slit lamp (Fig. 8A). The PBS group showed obvious iris hyperemia, synechia, and exudates in the anterior chamber. Compared with the PBS group, the eyes instilled with all the DEX formulations relieved the signs of inflammation. Most importantly, the DEX 10% SC-NEs group exhibited the weakest inflammatory response among all the DEX treatment groups. Furthermore, histopathological observation showed a large number of inflammatory cells in the iris-ciliary body from the PBS group (Fig. 8B). On the contrary, the DEX treatment groups, especially the DEX 10% SC-NEs group reduced infiltration of inflammatory cells. Finally, the cytokines mRNA expression levels in the iris-ciliary body were determined by RT-PCR (Fig. 8C). Compared with the PBS group, all the DEX treatment groups significantly lowered the MCP-1, MMP-1 and VCAM-1 mRNA levels. Moreover, the DEX 10% SC-NEs group exhibited the lowest mRNA levels of MCP-1, MMP-1 and VCAM-1 among the DEX treatment groups, indicating DEX 10% SC-NEs could significantly mitigate intraocular inflammation. In conclusion, all the results indicated that DEX SC-NEs greatly improved the in vivo anti-inflammatory efficacy in the EIU model due to the increased intraocular drug bioavailability.

### Ocular safety evaluation of SC-NEs in HCECs and rabbit eyes

**Fig. 9:**
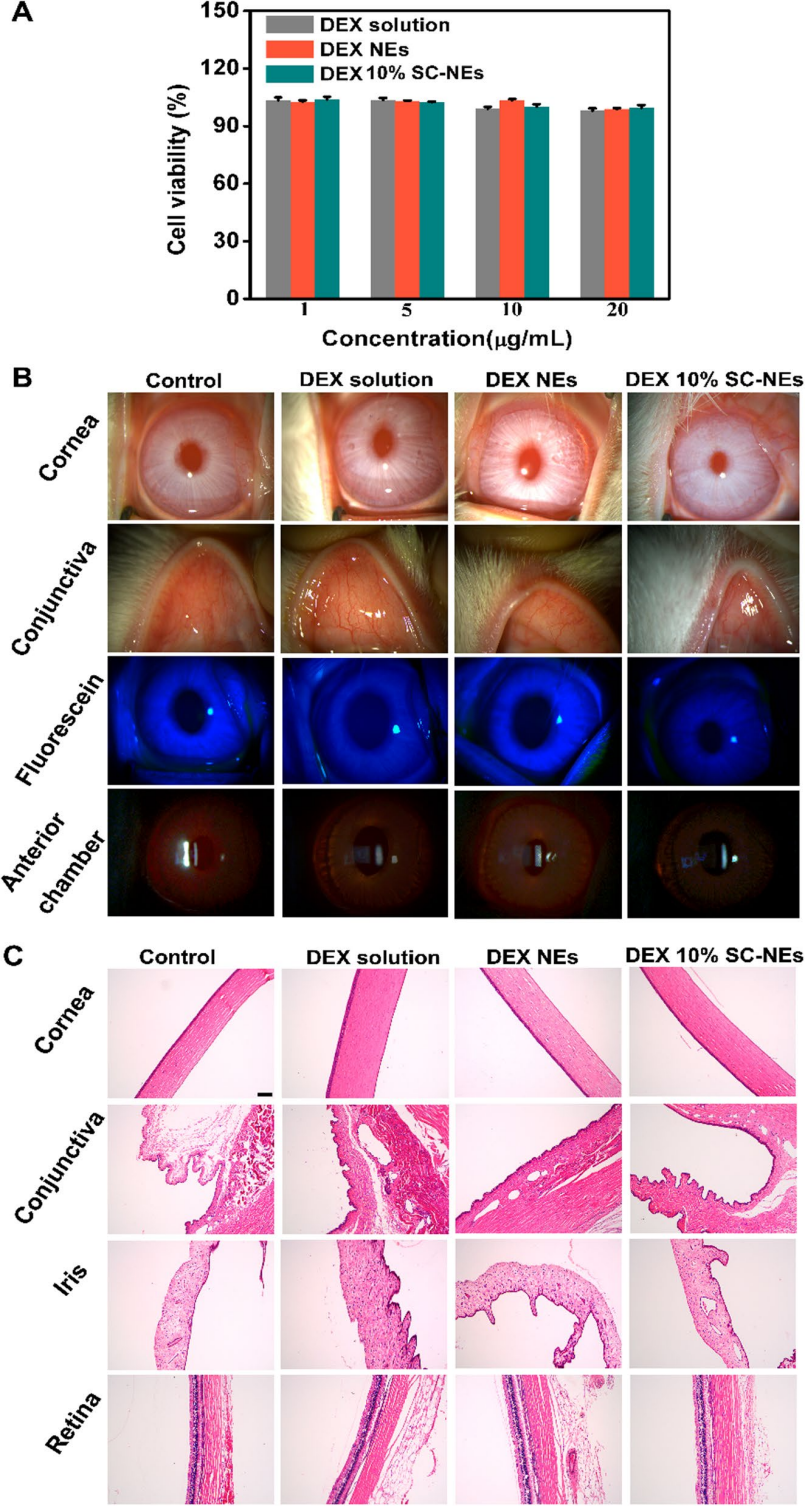
Ocular safety evaluation of DEX SC-NE in HCECs and rabbit eyes. Cytotoxicity test of DEX solution, DEX NEs, and DEX 10% SC-NEs in HCECs after 24 h of incubation (**A**) (n = 3); Ocular irritation observation after topical instillation of DEX solution, DEX NEs, and DEX 10% SC-NEs (**B**); HE staining observation of ocular tissues (**C**) (scale bar = 100 μm)

As an ocular drug delivery system, it would be expected to have good cytocompatibility and low cytotoxicity. Herein, we investigated the in vitro cytotoxicity of DEX SC-NEs in HECEs by CCK8 assay. As shown in Fig. 9A, the cell viability of DEX solution, DEX NEs, and DEX SC-NEs was more than 90% at a DEX concentration range of 1–20 μg/mL, suggesting that all three formulations had good cytocompatibility. Besides, there was no significant difference in the cell viability between DEX NEs and DEX SC-NEs, indicating that SC modification did not influence the cytotoxicity of NEs.

The in vivo ocular irritation of DEX SC-NEs was evaluated by the Draize test as previously reported [30]. No cornea and conjunctiva damage was observed by slit lamp after instillation of all three formulations (Fig. 9B). The result of the fluorescein staining test further confirmed the integrity of corneal epithelium (Fig. 9B). Besides, the Tyndall phenomenon was not observed in the anterior chamber, indicating no inflammatory response happened in the anterior chamber (Fig. 9B). Subsequently, the pathological changes of ocular tissues were evaluated by H&E staining. As shown in Fig. 9C, no morphology, structure alteration, and inflammatory cell infiltration were found in the cornea, conjunctiva, iris, and retina. These results manifested the better biocompatibility and ocular safety of DEX SC-NEs.

## Conclusion

We developed novel stearoyl L-carnitine-modified nanoemulsions that could target OCTN2 and ATB^0,+^ on the corneal epithelial cells for improving ocular drug delivery. A cellular uptake study in HCECs indicated that 10% of stearoyl L-carnitine-modified nanoemulsions could achieve optimal cellular uptake efficiency and the targeted nanoemulsions could transport across the cells dominantly via clathrin-, caveolin-mediated endocytosis, and macropinocytosis. In addition, an in vivo study suggested that the targeted nanoemulsions could significantly enhance trans-corneal permeation ability, prolong the ocular surface retention time, and improve ocular bioavailability. Most importantly, the targeted nanoemulsions significantly enhanced anti-inflammatory effects of dexamethasone compared with common solution and nanoemulsions in a rabbit model of EIU. Furthermore, the targeted nanoemulsions exhibited good biocompatibility in HCECs and ocular tissues. In summary, the stearoyl L-carnitine-modified nanoemulsions have great potential in nanomedicine for the treatment of ocular diseases.

### Supplementary Information

**Additional file 1: Fig. S1.** Characterization of stearoyl L-carnitine by ^1^H-NMR spectrum in CD_3_OD. **Fig. S2.** Characterization of stearoyl-L-carnitine by MS. **Fig. S3.** Solubility of DEX in oils, including medium-chain triglycerides (MCT), soybean oil, olive oil, and Labrafil® M1944 CS. **Table S1.** Effect of Labrafil® M1944 CS content on the physicochemical properties of DEX NEs. **Table S2.** Physicochemical characterization of SC-NEs. **Fig. S4.** The drug content changes of different NEs after storage at 4 °C. **Table S3.** Primers used for qRT-PCR.

## Data Availability

The data used in this article are available from the first authors on reasonable request.
