# Supplementary material for: OCTN2- and ATB0,+-targeted nanoemulsions for improving ocular drug delivery
Source: J Nanobiotechnology. 2024 Mar 26;22:130. doi: 10.1186/s12951-024-02402-x (PMC10964573; doi:10.1186/s12951-024-02402-x)
Supplement: Supplementary file 1 — Additional file 1: Fig. S1. Characterization of stearoyl L-carnitine by 1H-NMR spectrum in CD3OD. Fig. S2. Characterization of stearoyl-L-carnitine by MS. Fig. S3. Solubility of DEX in oils, including medium-chain triglycerides (MCT), soybean oil, olive oil, and Labrafil® M1944 CS. Table S1. Effect of Labrafil® M1944 CS content on the physicochemical properties of DEX NEs. Table S2. Physicochemical characterization of SC-NEs. Fig. S4. The drug content changes of different NEs after storage at 4 °C. Table S3. Primers used for qRT-PCR. [file 12951_2024_2402_MOESM1_ESM.docx]

**Additional file**

**OCTN2 and ATB^0,+^-targeted nanoemulsions for improving ocular drug delivery**

Bo Tang^1a^, Qiuxiang Wang^1a^, Guowei Zhang^2a^, Aiwen Zhang^1^, Lu Zhu^1^, Rongrong Zhao^1^, Hongwei Gu^2^, Jie Meng^2^, Junfang Zhang^2^**, Guihua Fang^1^*

^1^School of Pharmacy, Nantong University, 19 Qixiu Road, Nantong, Jiangsu Province, 226001, China

^2^Eye Institute, Affiliated Hospital of Nantong University, Nantong, Jiangsu Province, 226001, China

* Corresponding author: Guihua Fang (fangguihua@ntu.edu.cn)

**Corresponding author: Junfang Zhang ([junfangzh@126.com](mailto:junfangzh@126.com))

^a^ These authors contributed equally to this work

**1. Synthesis and Characterization of stearoyl L-carnitine**

First, L-carnitine and benzyl bromide were dissolved in N, N-Dimethylformamide (DMF) and continuously agitated at 130 °C for 4 h. The obtained mixtures were evaporated using vacuum drying at room temperature. The residue was purified by column chromatography to obtain L-carnitine benzyl ester. Second, stearic acid was dissolved in a flask containing dichloromethane (DCM). Oxalyl chloride and DMF were added dropwise into the flask. After agitating at room temperature for 2 h, the resultant mixture was concentrated under vacuum drying and the residue was dissolved in acetonitrile. L-carnitine benzyl ester was added to the reaction flask and agitated at 50 °C overnight. The resultant mixtures were evaporated and purified by column chromatography to obtain stearoyl L-carnitine benzyl ester. And finally, 10 % Pd/C was added into a methanol solution containing stearoyl L-carnitine benzyl ester and agitated at room temperature under a nitrogen atmosphere for 4 h. The reaction mixtures were filtered and followed by evaporating under a vacuum. The residue was purified by column chromatography to obtain stearoyl L-carnitine. The structure of stearoyl L-carnitine was confirmed by proton nuclear magnetic resonance (^1^H-NMR) (**Fig. S1**) and mass spectrum (MS) **(Fig. S2).**


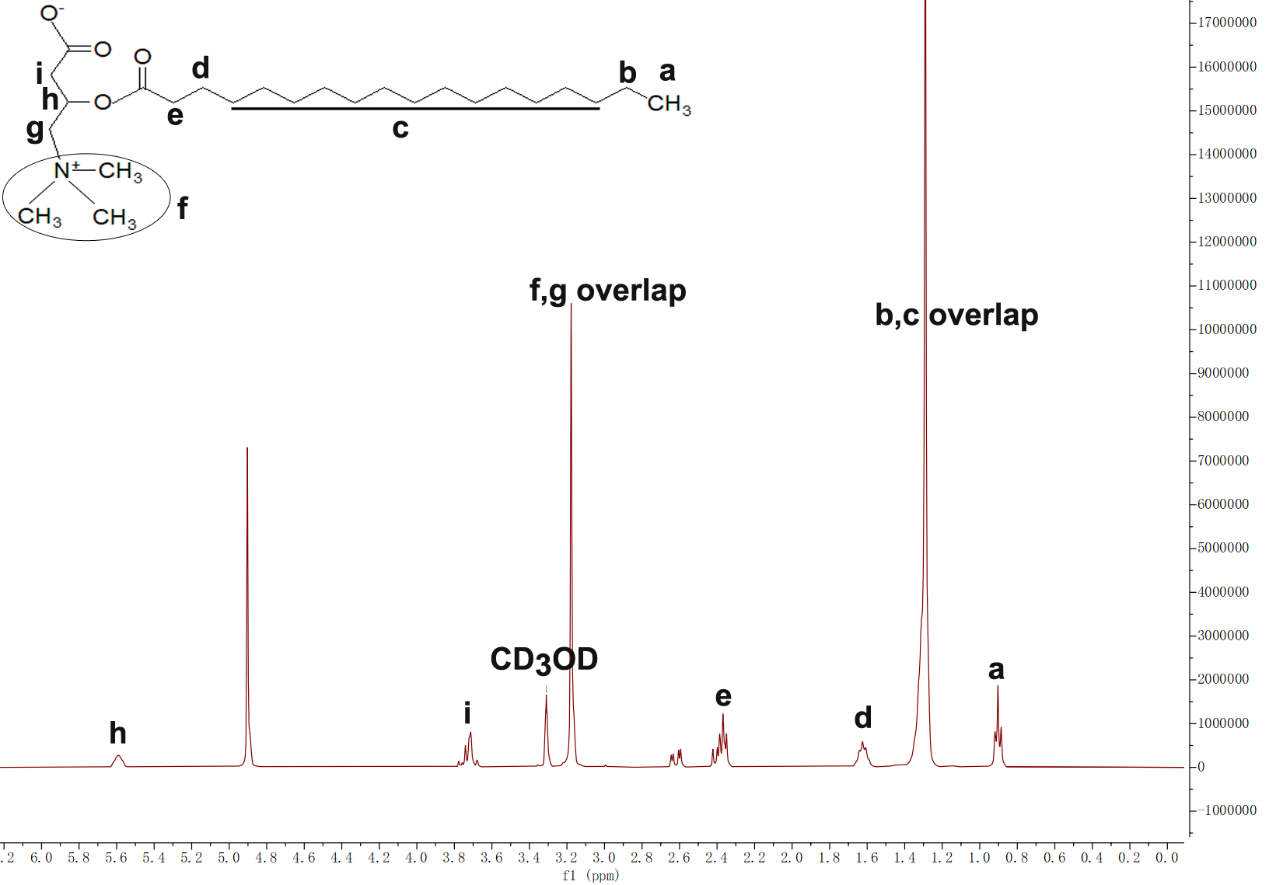


**Fig. S1** Characterization of stearoyl L-carnitine by ^1^H-NMR spectrum in CD_3_OD.

**

**

**Fig. S2** Characterization of stearoyl-L-carnitine by MS.

**2. Formulation development of Dexamethasone-loaded nanoemulsions (DEX NEs)**

The solubility of drug in oils is the basis for the selection of the oil phase. The solubility of DEX in various oils, including medium-chain triglycerides, soybean oil, olive oil, and Labrafil^®^ M1944 CS was determined by the shake flask method. Briefly, an excessive amount of DEX was added to 1mL of oil in an Eppendorf tube and mixed in a water bath sonication. Then the tubes were placed in a water bath shaker at 37 ± 0.5 °C. After shaking for 72 h, the samples were centrifuged at 10,000 rpm for 10 min. The supernatant was extracted with ethanol solution by sonicating for 20 min and centrifuging at 10,000 rpm for 10 min. The DEX concentration in the supernatant was determined by high-performance liquid chromatography. The DEX solubility in oils is shown in **Fig. S3**, DEX had a high solubility in Labrafil^®^ M1944 CS, so Labrafil^®^ M1944 CS was selected as the oil phase for further study. Next, the effect of the amount of Labrafil® M1944 CS content on the physicochemical properties of the DEX NEs was screened (**Table S1**). When the amount of Labrifil® M 1944 CS was 50 mg, DEX NEs had higher drug content (0.64±0.003mg/mL) and EE (91.3±2.8%), and this formulation was selected to further prepare DEX SC-NEs.


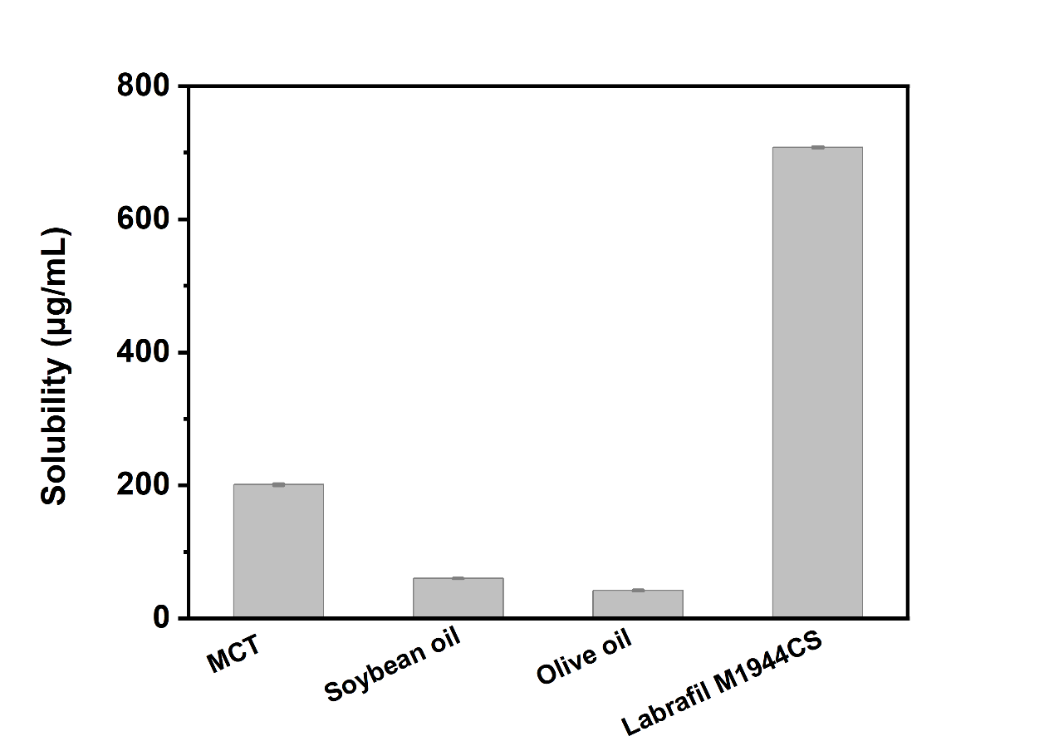


**Fig. S3** Solubility of DEX in oils, including medium-chain triglycerides (MCT), soybean oil, olive oil, and Labrafil^®^ M1944 CS.

**Table S1** Effect of Labrafil^®^ M1944 CS content on the physicochemical properties of DEX NEs.

| Labrafil 1944CS (mg) | Tween 80  (mg) | DEX Content (mg/mL) | Size(nm) | EE (%) |
| --- | --- | --- | --- | --- |
| 25 | 50 | 0.46±0.040 | 27.5±0.49 | 94.2±1.3 |
| 50 | 50 | 0.64±0.003 | 60.7±0.82 | 91.3±2.8 |
| 100 | 50 | 0.64±0.020 | 77.2±0.41 | 87.5±1.7 |

**Table S2** Physicochemical characterization of SC-NEs

| Formulations | Size(nm) | zeta potential (mV) | EE% |
| --- | --- | --- | --- |
| DEX NEs | 60.7±0.82 | -14.24±0.14 | 91.3±2.8 |
| DEX 5% SC-NEs | 62.1±0.58 | -14.01±0.13 | 93.7±1.1 |
| DEX 10% SC-NEs | 67.5±0.66 | -14.16±0.17 | 92.4±2.6 |
| DEX 20% SC-NEs | 69.4±0.44 | -13.97±0.18 | 91.2±1.3 |
| DEX 40% SC-NEs | 75.1±1.17 | -14.06±0.21 | 92.7±2.1 |
| DEX 80% SC-NEs | 75.5±0.94 | -14.32±0.28 | 90.8±1.1 |

**

**

**Fig.S4** The drug content changes of different NEs after storage at 4 °C.

**Table S3** Primers used for qRT-PCR

| Gene | Forward (5’-3’) | Reverse (5’-3’) |
| --- | --- | --- |
| MCP1 | GTCTCTGCAACGCTTCTGTGCC | AGTCGTGTGTTCTTGGGTTGTGG |
| MMP1 | TCAGTTCGTCCTCACTCCAG | TTGGTCCACCTGTCATCTTC |
| VCAM1 | GAACACTCTTACCTGTGTACAGC | CCATCCTCATAGCAATTAATGTGAG |
| GAPDH | GCGCCTGGTCACCAGGGCTGCTT | TGCCGAAGTGGTCGTGGATGACCT |
